# Ionotropic Receptors as Potential Targets Against Insect-Transmitted Diseases

**DOI:** 10.3390/biom16010076

**Published:** 2026-01-03

**Authors:** João Pessoa

**Affiliations:** Department of Medical Sciences and Institute of Biomedicine—iBiMED, University of Aveiro, 3810-193 Aveiro, Portugal; joao.pessoa@ua.pt

**Keywords:** insect, temperature, neuron, ion channel

## Abstract

Insects can remotely detect human temperature, odor, and other stimuli as part of their host-seeking strategy. Such detection involves specific biomolecules, whose inhibition could limit host spotting and decrease the spread of insect-transmitted diseases. In this framework, invertebrate-specific ionotropic receptors (IRs) provide a potential molecular target to disable the insect’s capability to detect stimuli from prospective hosts. While several IRs have been studied in disease-transmitting insects, their inhibition remains unexplored. The rational design and development of such inhibitors requires the detailed characterization of the structure and functional mechanisms of IRs. Here, I discuss a possible, exploratory, and long-term approach for IR inhibition, which is based on research in mammalian thermosensitive transient receptor potential ion channels.

## 1. Introduction

Throughout human history, insecticides have been fundamental to controlling the spread of insect-transmitted diseases, with benefits to both human health and agricultural yields. However, it is essential to recognize the impact of massive insecticide usage on both the health of humans and other nontarget species, as well as on the environment. Most currently used insecticides function by disrupting the nervous system, metabolism, or the growth of insects [1]. However, due to their high genetic variation rates, insects can adapt themselves to the stress caused by insecticides, which results in the requirement of increased insecticide dosages [2]. In these situations, insecticide replacement is also a necessary measure, which requires the development of novel insecticides or other chemical compounds to limit the negative effects of insects on their hosts.

**Figure 1:**
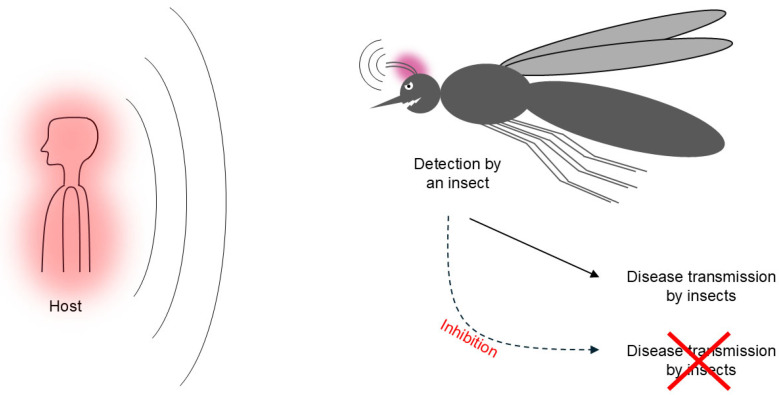
Detection of human stimuli by an insect. Through their antennae and other organs, insects can remotely detect the temperature, odor, and other stimuli (represented as a red glow) from humans and other potential hosts. Such detection allows it to find hosts and contaminate them with insect-carried diseases. Inhibiting the insects’ capability to detect such stimuli might limit the spread of these diseases. The insect’s anatomy is illustrative and not intended to be accurate.

Insects can remotely detect multiple stimuli from a potential host. Female mosquitoes can be attracted by exhaled carbon dioxide, body odors, silhouette, heat, and humidity of the host [3]. Disabling their temperature detection mechanisms could limit their disease-spreading capabilities [4] (Figure 1). Stimulus detection by insects involves the activation of specific ion channels [5]. Consistently, some insecticide targets are ion channels, including transient receptor potential (TRP) ion channels [6,7]. Nevertheless, another class of insect stimulus-sensitive biomolecules has been studied: ionotropic receptors (IRs) [8], which are an invertebrate-specific subtype of ionotropic glutamate receptors [9]. Each IR is heteromeric and is composed of a stimulus-specific tuning receptor and a more commonly expressed co-receptor, both corresponding to different subunits of the same protein [8,10]. IRs are involved in the detection of temperature, odor, taste, and humidity [9]. As such, they provide additional potential anti-insect targets, with the advantage of being invertebrate-specific.

IR inhibition has already been proposed as a strategy to decrease insect bites [9]. In the present article, I highlight the relevance of IRs in some disease-transmitting insects and provide a theoretical framework and potential approach for their inhibition.

## 2. Insect Ionotropic Receptors

**Figure 2:**
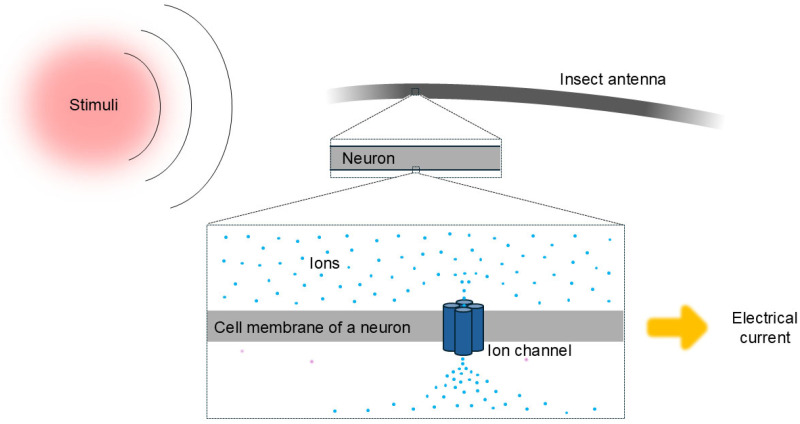
Molecular basis of host stimulus detection by an insect. In their antennae, insects contain neurons, which have ion channels inserted in their membrane. When an ion channel is crossed by ions, it generates an electrical current. Host stimuli alter the structure of the ion channel, affecting the ion flow and the resulting electrical current. The ion channel structural model and the direction of ion flow are illustrative and not intended to be accurate.

Insects detect temperature and other external stimuli mostly through their antennae, which have highly sensitive neurons [11]. These neurons contain ion channels (including IRs) that are sensitive to temperature, odor, humidity, and other stimuli from potential hosts (Figure 2).

*Drosophila* is an important model organism to study IRs and the expression of their respective coding genes [12]. In *Drosophila melanogaster* larva, the detection of cold temperatures is mediated by the expression of the genes coding for the IR21a tuning receptor (*Ir21a*) and its IR25a co-receptor (*Ir25a*) [13]. In adult *D. melanogaster*, the IR93a co-receptor functions in conjunction with IR21a and IR25a to mediate the response to the cold [14]. In *D. melanogaster*, the expression of at least three IR co-receptor-coding genes is involved in odor detection: *Ir25a*, *Ir8a*, and *Ir76b*. Among these, at least *Ir25a* is conserved in other insects, *Drosophila sechellia* and *Anopheles coluzzii* [15].

IRs have been identified in multiple mosquito species that can infect human hosts [8], including *Anopheles sinensis*, which transmits malaria and lymphatic filariasis [16]. At least seven IRs are expressed at their antennae [17]. In *Rhodnius prolixus*, a vector of Chagas disease, several IRs are expressed in their antennae and other sensory organs [18], in which the *Ir75a* tuning receptor-coding gene is the most abundantly expressed [19].

In *Aedes aegypti*, which transmits dengue, yellow fever, and chikungunya, many neurons express odor sensory receptors, bringing complexity to their sensing mechanisms [20]. In this mosquito, humidity detection involves the expression of the *Ir40a* and *Ir68a* tuning receptor-coding genes [21], as well as the *Ir93a* co-receptor-coding gene [22]. Furthermore, expression of the *Ir8a* co-receptor-coding gene is involved in chemoreception [23]. Expression of the *Ir140* tuning receptor-coding gene is involved in temperature detection and may compensate for the depletion of other sensory proteins [24]. Short-chain carboxylic acids can activate the expression of the *Ae. aegypti Ir75* tuning receptor-coding gene [25]. In addition, the expression of the *IR8a* co-receptor-coding gene mediates the detection and attraction to standing water [26] and is also involved in the detection of human odor [27].

Multiple IRs have been identified in the malaria vector *Anopheles gambiae* [28], many of which at the antennae [29]. In this mosquito, expression of the *Ir21a* tuning receptor-coding gene is an essential requirement for heat-seeking and heat-stimulated blood feeding [30]. Furthermore, expression of the *Ir93a* co-receptor-coding gene is also involved in the detection of heat and humidity [22]. Multiple IR-coding genes were identified in other malaria vectors, *An. Coluzzii*, and *Anopheles quadriannulatus* [31]. In *An. coluzzii*, expression of the *Ir76b* co-receptor-coding gene is involved in olfaction, blood feeding, and mating [32].

These examples demonstrate that IRs are important for several disease-transmitting insects to locate their potential hosts. Therefore, their inhibition would hold great promise against the spread of those diseases. The structural organization of IRs has already been uncovered [10]; however, their inhibition will require a detailed characterization of the structure and molecular mechanisms of these ion channels. For the achievement of such a goal, a seemingly unrelated group of ion channels, mammalian thermosensitive TRP channels [33], provides a possible strategy that could be pursued in IRs.

## 3. Mammalian Thermosensitive TRP Ion Channels

In mammals, temperature detection starts in neurons located beneath the skin, which contain thermosensitive ion channels inserted in their cell membrane. The structure of these ion channels is changed by temperature (or temperature variations), which modifies the electrical currents generated by these channels. These electrical currents are propagated to the brain via the spinal cord. In the brain, electrical currents are interpreted as temperatures or temperature changes [4] (Figure S1). In mammals and other organisms, TRP ion channels are important in the detection of temperature.

Technical advances in cryo-electron microscopy (cryo-EM) have catalyzed the determination of structures for several mammalian TRP channels [34]. In some of these structures, the TRP channel is inserted in a nanodisc, a membrane nanoparticle that provides a native-like environment for a membrane protein [35]. Several structures have been determined using the heat-sensitive channels TRP melastatin 2 (TRPM2), TRP melastatin 4 (TRPM4), and TRP vanilloid 3 (TRPV3). Structures of the human TRPM2 channel provided insights into its gating mechanism [36,37]. Comparison of structures of the human TRPM4 channel determined at 4 °C and 37 °C revealed its ‘warm’ and ‘cold’ conformations [38,39]. The comparison of structures of the mouse TRPV3 channel in its temperature-dependent open, closed, and intermediate states revealed conformational changes associated with different temperatures and contributed insights into its molecular mechanism of temperature detection [40,41]. An additional structure of the mouse TRPV3 channel also revealed insights into its gating mechanism [42]. A structure of the human TRP melastatin 8 (TRPM8) channel (activated by cold temperatures) has also been determined [43]. These structures have contributed insights into the functional mechanisms of thermosensitive TRP channels, encouraging similar studies in IRs.

The activity of several mammalian TRP channels can be modulated or inhibited. These channels can be regulated by phosphoinositides [44]. A potential druggable site was identified in the structure of the human cold-sensitive TRP ankyrin 1 (TRPA1) channel [45]. Two selective inhibitors of the human TRPM2 channel have been tested [46]. The rat heat-sensitive TRP vanilloid 1 (TRPV1) channel can be modulated by natural lipids [47]. Increasing tyrosine phosphorylation altered the chemical and thermal sensitivities of the rat heat-sensitive TRP vanilloid 2 (TRPV2) channel [48], which could also be modulated by chemical compounds [49]. There are several agonists and antagonists known for the human heat-sensitive TRP vanilloid 4 (TRPV4) channel [50]. The mouse cold-sensitive TRPM8 channel could also be desensitized or inhibited with chemical compounds [51]. These examples in mammalian thermosensitive TRP channels could encourage the development of chemical compounds for the modulation, desensitization, or inhibition of insect IRs. However, their development and effective application with disease control outcomes would be a long-term goal, facing multiple challenges, as discussed below.

## 4. Discussion

The present opinion article develops the previously proposed hypothesis that inhibiting IRs could limit the capability of insects to detect potential hosts [8,9]. A convenient feature of IRs is their exclusive location to invertebrates [8,9]. Therefore, their inhibition should not have any outstanding effects on other animals. To inhibit IRs, these biomolecules need to be structurally and mechanistically characterized to enable the rational design of specific inhibitors.

The first step in this process would be the selection of a few IRs for detailed structural and mechanistic studies. For this purpose, the ideal IR should have a critical biological function in stimulus detection in multiple disease-transmitting insects. Finding convenient IRs should be based on studies that demonstrate their in vivo function, including those mentioned in the present article. A detailed survey (including tuning receptors and co-receptors) should uncover the most essential IRs in their respective functional networks. After selecting candidate IRs, these membrane proteins would have to be purified, their structures determined, and their molecular mechanisms understood. Nanodiscs would be convenient platforms for structure determination, as they keep the native environment of membrane proteins. A major challenge in protein structure determination is its heterologous expression and purification in sufficient amounts. Nevertheless, the increasing utilization of cryo-EM in structural studies opens exciting opportunities because, unlike X-ray crystallography, cryo-EM requires low amounts of purified protein. Therefore, the feasibility of the determination of high-resolution structures has been increasing.

Based on the structures and molecular mechanisms of selected IRs, inhibitory strategies with chemical compounds would have to be developed. Potential inhibitors should be designed by computational approaches, synthesized, and tested through functional assays using purified IRs. Then, the inhibitors would have to be tested in living insects. Several studies have tested the effects of IR gene expression and silencing on insect behaviors that include seeking heat [24,30], blood [21], water [21,26], or odor [27]. These assays could be adapted to test the effect of IR inhibitors on these insect behaviors.

The structural and mechanistic characterization of IRs could be inspired by the approach that has already been used for mammalian thermosensitive TRP ion channels. Therefore, a similar strategy could be tested in IRs. Nevertheless, IRs and TRP channels have distinct evolutionary origins, ligand specificities, and gating mechanisms. Therefore, they may also differ in pharmacological druggability, despite their structural and functional similarities. Furthermore, suppressing specific host detection cues (through IR inhibition) could promote insect evolution, resulting in upgrades in other sensory ways, such as vision, mechanosensation, or others. It is also unclear whether IR inhibitors could affect non-target insects with important ecological roles. As such, any evolutionary and ecological risks should also be considered.

Since IRs are involved in the detection of temperature, odor, taste, and humidity [9], their inhibition in insects should affect their detection of multiple stimuli, resulting in a potent multifactorial approach to disabling the insect’s capability to detect potential hosts. Of note, in *An. coluzzii*, at least one IR is involved in mating [32], whose inhibition might also challenge the proliferation of this mosquito. Since IR inhibitors must not be harmful to non-insect animals, they would also have to be tested in mammalian and other vertebrate animal models. IR inhibitors should be applicable by spraying and should also have minimal environmental impact and polluting effects. In the framework of IR inhibitor delivery, their volatility, chemical stability, and efficiency of uptake by insects would need to be assessed. Their selectivity among different IR subtypes would also be a critical aspect. To optimize the timings of IR inhibitor delivery, the life cycles of the target insects should be considered. Furthermore, spatial restrictions on spraying, rather than its large-scale, should minimize any impacts on beneficial and non-target organisms. Importantly, they should not be used on pollinating insects (or any other insects with an ecological niche) to prevent any ecosystem disruption. IR inhibitors would also have to be subjected to ethical and regulatory legislation.

Currently, there are no known broadly effective inhibitors of insect IRs [8]. As such, it is unclear whether they could be species-specific or active against multiple species. It is also unknown if IRs can actually be inhibited, if such inhibition would significantly affect the insect’s host-seeking behavior, or if it would be selective and without off-target effects. Therefore, disease control through effective IR inhibitors is currently a hypothetical and long-term goal. Nevertheless, their discovery could be a valuable step to decrease insect bites and bring benefit to humankind.

## 5. Conclusions

IRs are prospective drug targets for disabling insects’ capability to detect potential hosts through their temperature, odor, mating, and other stimuli. Inhibiting these invertebrate-specific biomolecules could be a new approach to limit the spread of insect-transmitted diseases. It would also be a challenging and time-consuming endeavor. Importantly, the rational design of IR inhibitors will require detailed structural and mechanistic characterization of IRs, which might benefit from the approach already in use for mammalian thermosensitive TRP ion channels.

## Data Availability

No new data were created or analyzed in this study. Data sharing is not applicable to this article.

## Supplementary Materials

The following supporting information can be downloaded at: https://www.mdpi.com/article/10.3390/biom16010076/s1, Figure S1: Transmission of a temperature stimulus into the human brain.

## Abbreviations

The following abbreviations are used in this manuscript:

IR	Ionotropic receptor
TRP	Transient receptor potential
TRPA	Transient receptor potential ankyrin
TRPM	Transient receptor potential melastatin
TRPV	Transient receptor potential vanilloid
