# Supplementary material for: Ionotropic Receptors as Potential Targets Against Insect-Transmitted Diseases
Source: Biomolecules. 2026 Jan 3;16(1):76. doi: 10.3390/biom16010076 (PMC12838988; doi:10.3390/biom16010076)
Supplement: Supplementary file 1 [file biomolecules-16-00076-s001.zip › biomolecules-4031807-supplementary.pdf]

Ionotropic receptors as potential targets against insect-transmitted diseases

João Pessoa

Department of Medical Sciences and Institute of Biomedicine—iBiMED, University of Aveiro, 3810-193 Aveiro, Portugal; joao.pessoa@ua.pt

Supplementary Information

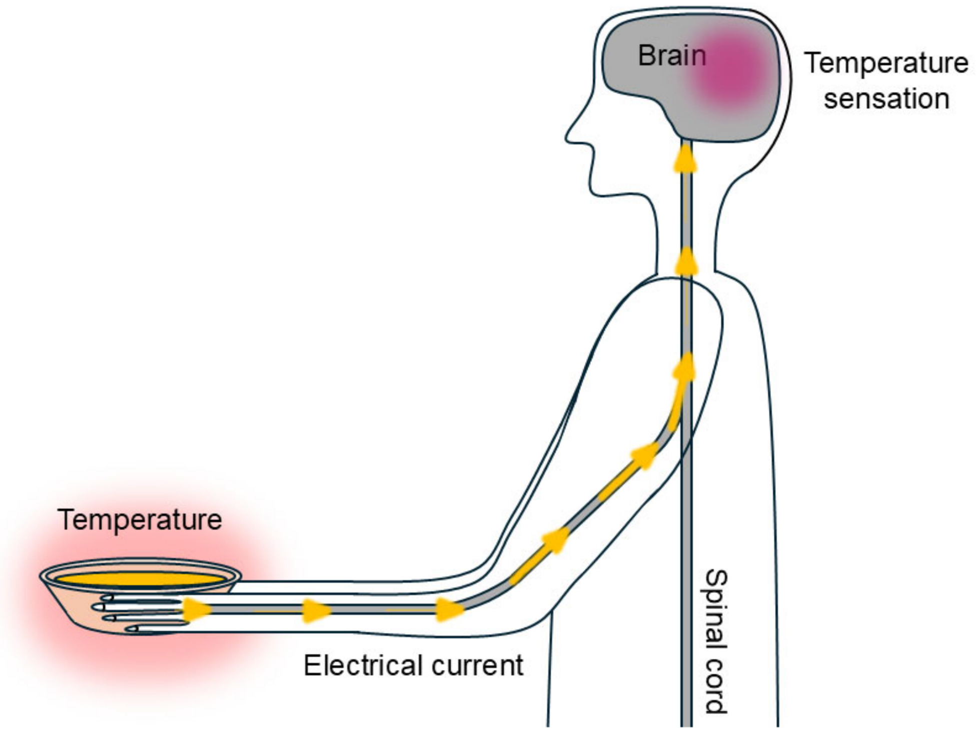

Figure S1. Transmission of a temperature stimulus into the human brain. Temperature affects the electrical currents produced by neurons. These electrical currents are transmitted through bundles of neurons connected to the spinal cord (both represented in gray). The spinal cord delivers the electrical currents into the brain, where they are decoded into a temperature sensation (represented in purple, in the brain).
